# Antartin, a Cytotoxic Zizaane-Type Sesquiterpenoid from a *Streptomyces* sp. Isolated from an Antarctic Marine Sediment

**DOI:** 10.3390/md16040130

**Published:** 2018-04-16

**Authors:** Dayoung Kim, Eun Ju Lee, Jihye Lee, Alain S. Leutou, Yern-Hyerk Shin, Bomi Choi, Ji Sun Hwang, Dongyup Hahn, Hyukjae Choi, Jungwook Chin, Sung Jin Cho, Yong Deog Hong, Jaeyoung Ko, Chi Nam Seong, Katherine N. Maloney, Dong-Chan Oh, Inho Yang, Hayoung Hwang, Sang-Jip Nam

**Affiliations:** 1 Department of Chemistry and Nano Science, Ewha Womans University, Seoul 03760, Korea; rlaekdud0503@naver.com (D.K.); jl3414@gmail.com (J.L.); leutoualain@yahoo.fr (A.S.L.); bomeec87@naver.com (B.C.); 2 New Drug Development Center, Daegu-Gyeongbuk Medical Innovation Foundation (DGMIF), Daegu 41061, Korea; ejlee@dgmif.re.kr (E.J.L.); hjs1228@dgmif.re.kr (J.S.H.); jwchin@dgmif.re.kr (J.C.); sjcho@dgmif.re.kr (S.J.C.); 3 Natural Products Research Institute, College of Pharmacy, Seoul National University, San 56-1, Sillim, Gwanak, Seoul 08826, Korea; itsue00@snu.ac.kr (Y.-H.S.); dongchanoh@snu.ac.kr (D.-C.O.); 4 School of Food Science and Biotechnology, College of Agriculture and Life Sciences, Kyungpook National University, Daegu 41566, Korea; dohahn@knu.ac.kr; 5 Institute of Agricultural Science & Technology, Kyungpook National University, Daegu 41566, Korea; 6 College of Pharmacy, Yeungnam University, Gyeongsan-si, Gyeongsangbukdo 38541, Korea; h5choi@yu.ac.kr; 7 Materials Lab Amorepacific R&D Unit, Yongin, Gyeonggi-do 17074, Korea; hydhong@amorepacific.com (Y.D.H.); jaeyoungko@amorepacific.com (J.K.); 8 Department of Biology, College of Life Science and Natural Resource, Sunchon National University, Suncheon 57922, Korea; scnu@sunchon.ac.kr; 9 Department of Chemistry, Point Loma Nazarene University, 3900 Lomaland Drive, San Diego, CA 92106, USA; KatherineMaloney@pointloma.edu; 10 Department of Convergence Study on the Ocean Science and Technology, Korea Maritime and Ocean University, Busan 49112, Korea

**Keywords:** *Streptomyces* sp., cold water natural product, marine natural product, zizaane-type sesquiterpenoid

## Abstract

Antartin (**1**), a new zizaane-type sesquiterpene, was isolated from *Streptomyces* sp. SCO736. The chemical structure of **1** was assigned from the interpretation of 1D and 2D NMR in addition to mass spectrometric data. The relative stereochemistry of **1** was determined by analysis of NOE data, while the absolute stereochemistry was decided based on a comparison of experimental and calculated electronic circular dichroism (ECD) spectra. Antartin (**1**) showed cytotoxicity against A549, H1299, and U87 cancer cell lines by causing cell cycle arrest at the G1 phase.

## 1. Introduction

Sesquiterpenes comprise an important class of natural products with diverse bioactivities including antibacterial, antifungal, antiviral, antitumor, cytotoxic, and immunosuppressive activities [1,2,3,4,5,6]. As secondary metabolites, sesquiterpenes are commonly produced from terrestrial organisms. However, the occurrence of marine sesquiterpenes is expanding along with the growing importance of marine natural products in drug discovery.

Marine organisms are considered a fruitful source of novel chemical structures possessing a variety of biological activities [7,8]. Yet, of the more than 25,000 marine natural products [9] that have been described, less than 3% were discovered from cold-water habitats, a fact attributed to limited physical accessibility [10,11]. However, the last 10 years have seen remarkable progress in the discovery of cold-water-derived marine natural products [10,12,13]. In particular, the proportion of new marine natural products coming from cold-water-derived microbes had increased from 22% to 71% during this period [10].

**Figure 1:**
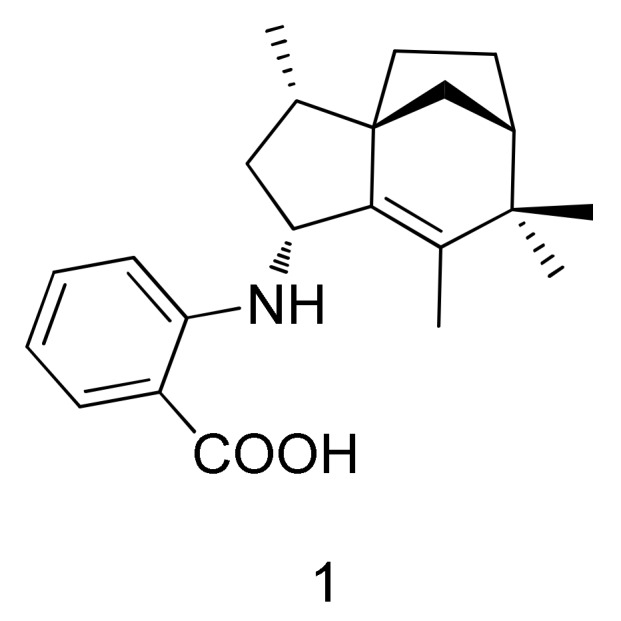
Structure of compound **1**.

As part of our investigation of novel bioactive marine natural products from cold-water-derived bacteria, a new zizaane-type sesquiterpene was isolated from *Streptomyces* sp. SCO-736, originating from an Antarctic marine sediment. Herein, we report the isolation and structure elucidation of antartin (**1**) with its biological activity (Figure 1).

## 2. Results and Discussion

### 2.1. Isolation and Structure Elucidation

**Figure 2:**
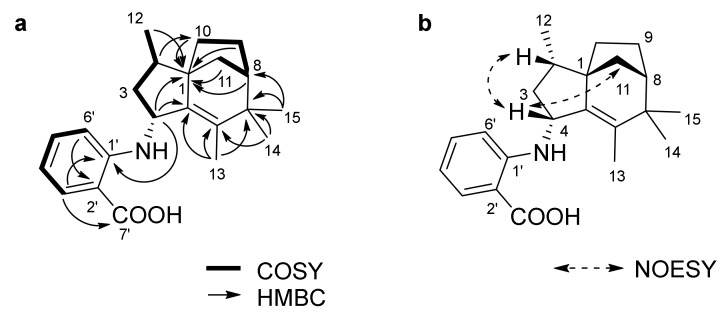
COSY and key HMBC correlations (**a**), and NOESY correlations (**b**) of antartin (**1**).

Antartin (**1**) was obtained as a brown solid. The high resolution fast-atom bombardment mass spectrometry (HRFABMS) spectrum of compound **1** showed an [M]^+^ ion peak at *m*/*z* 339.2196, which suggested the molecular formula C_22_H_29_NO_2_. This molecular formula accounted for nine degrees of unsaturation. The IR spectrum of **1** showed the presence of a secondary amine (3365 cm^−1^) and a carboxylic acid group (1660 and 2936 cm^−1^). The ^1^H NMR spectrum of **1** displayed one nitrogenated methine proton (δ_H_ 4.24 (1H, d, *J* = 6.1 Hz)), one doublet (δ_H_ 0.94 (3H, d, *J* = 6.8 Hz)), and three methyl singlets (δ_H_ 1.04 (3H, s), δ_H_ 1.10 (3H, s), δ_H_ 1.52 (3H, s)). The ^1^H, ^13^C, and heteronuclear single quantum coherence (HSQC) spectroscopic data revealed four methyl, seven methine, four methylene, one carbonyl, and six quaternary carbons (Table 1). Interpretation of 2D NMR spectroscopic data allowed us to put together the structure of **1**. The ^1^H-^1^H correlation spectroscopy (COSY) crosspeaks (H-4/H-3a/H-2/H-12 and H-10/H-9/H-8/H-11) illustrated two spin systems, each composed of four carbon units (C-4/C-3/C-2/C-12 and C-10/C-9/C-8/C-11). Heteronuclear multiple-bond correlation (HMBC) correlations from H-13 to C-5/C-6/C-7, from H-14 to C-6/C-7, and from H-15 to C-7/C-8 allowed us to connect C-5/C-6/C-7/C-8. Furthermore, three-bond HMBC correlations from H-8/H-9a/H-12 to C-1, and from H-2 to C-10, as well as long-range HMBC correlations from H-4 to C-1/C-5 revealed the connectivity of C-4/C-5/C-1/C-10, which completed the construction of a zizaane-type 5/6/5 bridged sesquiterpene ring structure. The COSY crosspeaks (H-3′ (1H, dd, *J* = 6.9, 2.0 Hz)/H-4′ (1H, dd, *J* = 6.9, 6.9 Hz)/H-5′ (1H, dd, *J* = 6.9, 6.9 Hz)/H-6′ (1H, dd, *J* = 6.9, 2.0 Hz)) revealed a 1,2 disubstituted benzene ring moiety, which was connected to C-4 through NH based on the observation of an HMBC correlation from H-4 to C-1′ and on the carbon chemical shift of C-4 (δ_C_ 53.9). Thus, the planar structure of antartin (**1**) was determined, as shown in Figure 2.

The relative configurations of **1** were determined by the analysis of nuclear Overhauser effect spectroscopy (NOESY) correlations. NOESY crosspeaks of H-11 and H-2 to H-4 indicated that the protons H-11, H-4, and H-2 were located on the same face of the tricycle ring.

Compound **1** is a member of the zizaane-type sesquiterpenoid family of natural products. This family caught the interest of synthetic organic chemists, and several asymmetric methods have been developed for the preparation of zizaane-type sesquiterpenoids [14,15]. However, these synthetic transformations are inherently difficult, and the absolute stereochemistry for naturally-occurring zizaane-type sesquiterpenoids was initially inferred on the basis of comparison of optical rotation values alone. In 2009, an X-ray crystal structure of albaflavenone was published, and its validated absolute configurations provided an important reference point for other members of this class of natural products [16]. In 2013, strepsesquitriol—a rearranged zizaane-type sesquiterpenoid—was the subject of a study employing quantum-chemical calculations to derive a theoretical optical rotation to reveal its stereochemistry [17].

**Figure 3:**
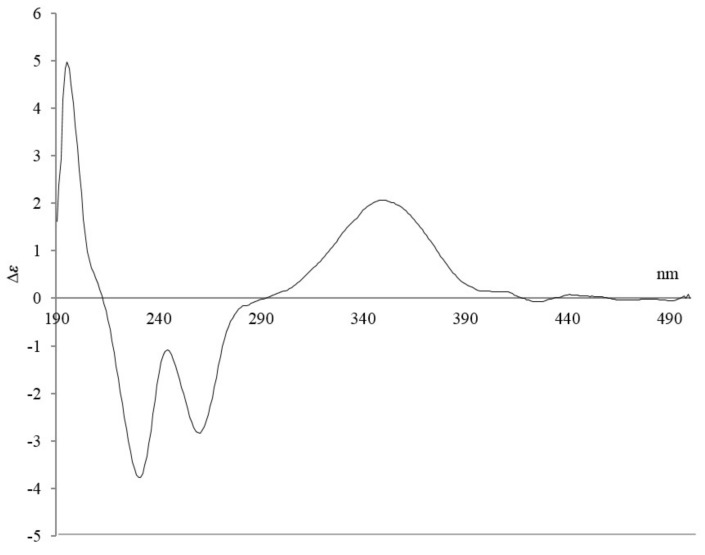
Electronic circular dichroism (ECD) spectrum for antartin (**1**).

Compound **1** possesses a strong chromophore derived from a benzene ring, thus making it possible to obtain an experimental electronic circular dichroism (ECD) spectrum. We compared the experimental ECD spectrum of **1** with the calculated ECD spectra using the density functional theory (DFT) model. The experimental ECD spectrum of **1** showed two distinct positive Cotton effects at 198 and 351 nm, as well as negative Cotton effects at 232 and 261 nm (Figure 3). While **1** contains four stereocenters (at C-1, C-2, C-4, and C-8) corresponding to 16 theoretically possible stereoisomers, the one-carbon bridge connecting C-1 to C-8 constrains the relative stereochemistry of these two stereocenters (to either 1*R*, 8*S* or 1*S*, 8*R*), leaving a total of eight possibilities. We calculated the ECD spectra for all eight stereoisomers, only one of which displayed the same patterns as the experimental ECD spectrum of **1** (Figure S1). This result supported the absolute configurations of 1*R*,2*S*,4*R*,8*S* for antartin (**1**, Figure 2b).

Plausible biosynthetic pathways for this class of natural products and other related bridged sesquiterpenoids have been suggested by Li et al. [18]. Compound **1** may share a biosynthetic pathway with related natural products epi-isozizaane [19] and (4*R*)-albaflavenol [20]. In 2013, strepsesquitriol, a rearranged zizaane-type sesquiterpenoid, was isolated, and the determined structure showed analogous orientations for C-2, C-4, and the bridge [17].

### 2.2. Bioactivities

**Figure 4:**
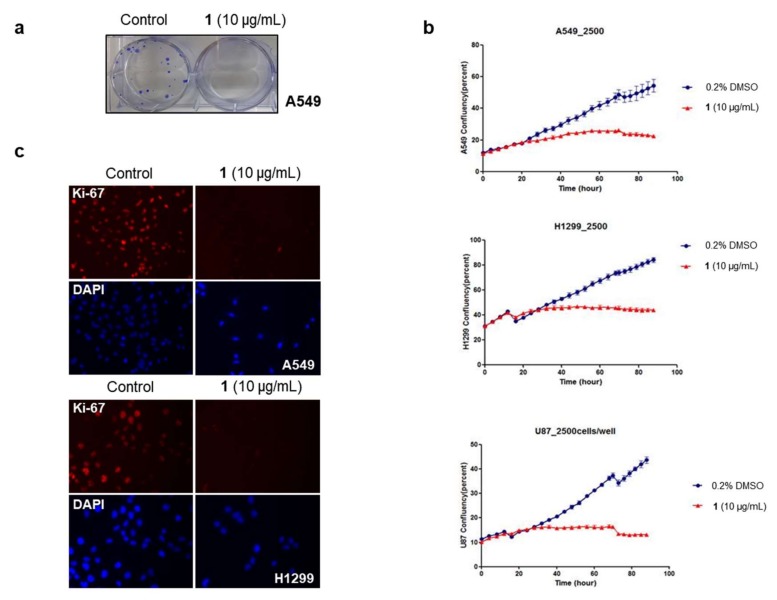
Effect of compound **1** on the growth of lung cancer cells and brain tumor cells. (**a**) Focus-forming assay. The absence of foci in the **1**-treated cells suggests that **1** suppressed lung cancer cell tumorigenic potential; (**b**) Cell growth assay. Cancer cell growth in real time was monitored for four days after treatment with **1**, showing no cell growth (Blue dot: 0.2% DMSO, Red dot: 10 μg/mL **1**); (**c**) Immunofluorescence staining experiment. In **1**-treated cells, the expression of Ki-67, a representative proliferation marker, disappeared.

In order to explore the biological activities of **1**, we performed a cellular proliferation assay using several cancer cell lines originating from diverse tumor types [21]. The results shown in Table 2 illustrate that **1** has a strong cytotoxic effect on cancer cells at a concentration of 20 μg/mL (59 μM). Among these, we selected the non-small cell lung carcinoma (NSCLC) cell lines and brain tumor cell line for further analysis based on research interests of solid tumor types, and determined the dose of **1** required for 50% growth inhibition (GI_50_) in those cells. GI_50_ values for **1** varied slightly between 4 and 8 μg/mL for different cancer cell types (Figure S8). Next, we performed a focus formation assay to determine whether **1** would inhibit the in vitro tumorigenic potential of cancer cells. A549 cells formed foci in an anchorage-dependent manner, while **1**-treated cells failed to form foci (Figure 4a). This result suggests that **1** may inhibit the tumorigenesis of solid lung tumor cells.

We observed cancer cell growth for four days in the presence or absence of **1**. As shown in Figure 4b, treated cells did not show an increase in cell numbers, instead maintaining initial cell confluency throughout the culture. Furthermore, immunofluorescence staining showed that the expression of Ki-67, an important marker of proliferation, was not detected in **1**-treated cells (Figure 4c). From these results, it can be postulated that **1** inhibits cancer cell growth by suppressing cell proliferation (i.e., arrest), rather than by activating a cell death mechanism (such as apoptosis or necrosis).

**Figure 5:**
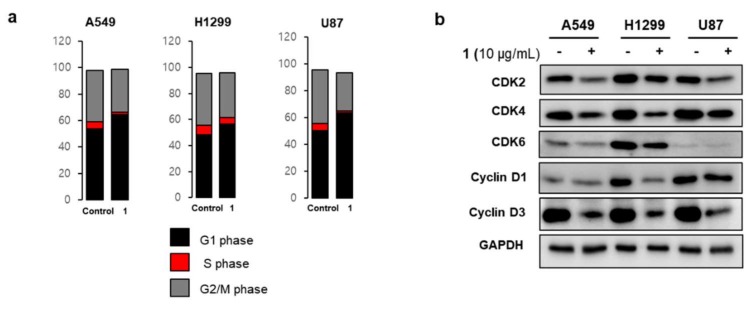
Cell cycle arrest by treatment with **1**. (**a**) Lung cancer cells (A549 and H1299) and brain tumor cells (U87) showed cell cycle arrest at the G1 phase after treatment with **1**; (**b**) Western blot analysis of cell cycle-associated proteins revealed that **1** downregulated the expressions of cell cycle transit proteins from the G1 to S phases, resulting in G1 arrest.

Based on the results of the cell confluency experiment, we decided to probe the cell cycle after treatment with **1** for 24 h, when difference in cell confluence between **1**-treated and DMSO-treated cells started to appear. Cell cycle arrest was analyzed by measuring DNA amounts in the G1, S, G2/M phases. Results shown in Figure 5a indicate that tested cancer cell lines induce G1 arrest by treatment with **1**, as DNA contents at the G1 phase increased and DNA contents at the S phase decreased. We next evaluated the effects of **1** on the expression of cell cycle check proteins, which are exclusively observed in the G1 phase transit to the S phase. Figure 5b showed that cell cycle-regulating proteins such as CDKs and cyclins were downregulated by treatment with **1** [22].

## 3. Materials and Methods

### 3.1. General Experimental Procedures

The optical rotation was measured using an Autopol III (Rudolph Research Analytical, Hackettstown, NJ, USA) polarimeter with a 5-cm cell. The UV spectrum was recorded in MeOH on a S-2100 (Scinco, Seoul, Korea). The ECD spectrum was recorded using an Applied Photophysics Chirascan-Plus circular dichroism spectrometer (Applied Photophysics Ltd., Leatherhead, Surrey, UK). The IR spectrum was collected on a Varian Scimitar Series. NMR spectra were obtained using a Varian Inova NMR spectrometer (Varian, Inc., Palo Alto, CA, USA; 500 and 125 MHz for ^1^H and ^13^C NMR, respectively), using the signals of the residual solvent as internal references (δ_H_ 3.31 and 4.78 ppm and δ_C_ 49.1 ppm for methanol-*d*_4_). High resolution mass spectrum was obtained on a JMS-700 (JEOL Ltd., Tokyo, Japan) mass spectrometer. Low-resolution LC-MS data were analyzed using an Agilent Technologies 6120 quadrupole LC/MS system with a reversed-phase column (Phenomenex Luna C18(2) 100 Å, 50 mm × 4.6 mm, 5 μm) at a flow rate of 1.0 mL/min. Column chromatography separation was performed using C18 (40–63 μm, ZEO prep 90), eluting with a gradient of methanol and water. The fractions were purified using a reversed-phase HPLC Watchers 120 ODS-BP (250 mm × 10 mm, 5 μm) column, eluting with 80% CH_3_CN in H_2_O at flow rate of 2.5 mL/min.

### 3.2. Strain Isolation and Fermentation

Actinomycete strain SCO736 was isolated from marine sediments collected off the coast of Antarctica. Strain SC0736 was assigned as a member of the genus *Streptomyces* sp., with 99.7% identity. The 16S rRNA gene sequence was deposited in GenBank (accession number F-BS033001). Actinomycete strain SC0736 was cultured in 20 × 2.5 L Ultra Yield Flasks, each containing 1 L of the medium (10 g/L soluble starch, 2 g/L yeast, 4 g/L peptone, 10 g/L CaCO_3_, 20 g/L KBr, 8 g/L Fe_2_(SO_4_)_3_·4H_2_O dissolved in 750 mL natural seawater and 250 mL of distilled water) at 25 °C with shaking at 150 rpm. After seven days, the broth was extracted with EtOAc (20 L overall) to afford 0.9 g of the organic extract.

### 3.3. Extraction and Purification

The extract was subjected to flash vacuum chromatography on C18 resin, eluting with a step gradient from 10 to 100% MeOH in H_2_O. The last fraction eluted with 100% MeOH (110.0 mg) was subjected to reversed-phase HPLC with 80% aqueous acetonitrile (Watchers 120 ODS-BP, 250 × 10 mm, 5 μm, 2.5 mL/min, UV = 210 nm) to afford antartin (**1**, 2.5 mg) with a retention time of 53 min.

Antartin (**1**): amorphous, brown solid; [α]^23^_D_ + 177 (*c* 0.2, MeOH); UV (MeOH) *λ*max (log ε) 220 (1.80), 260 (1.43), 340 (1.13) nm; IR (KBr) ν_max_ 3365, 2936, 2864, 2348, 1660, 1574, 1509, 1445, 1238, 910, 750 cm^−1^, ^1^H and ^13^C NMR data, see Table 1; HRFABMS *m*/*z* 339.2196 [M]^+^ (calcd for C_22_H_29_NO_2_, 339.2193).

### 3.4. Computational Analysis

The energy-minimized modeling of eight possible isomers of **1** was performed with density functional theory (DFT) calculations using Turbomole 6.5 (basis set: def-SV(P) for all atoms; functional: B3-LYP) to obtain ground-state geometries. The ECD spectra data from the calculated structures were acquired with the basis set def-SV(P) for all atoms at the DFT level, using the B3-LYP functional. The acquired ECD data were simulated by overlapping for each transition, where σ is the width of the band at 1/e height, and Δ*E_i_* and *R_i_* are the excitation energies and rotatory strengths for transition *i*. In this work, the value of σ was fixed at 0.10 eV (Table S1).

$$\Delta \epsilon \left(E\right)=\frac{1}{2.297\times 10^{-39}}\frac{1}{\sqrt{2\pi \sigma }}\overset{i}{\underset{A}{\sum }}\Delta E_{i}R_{i}e^{\left[-{\left(E-\Delta E_{i}\right)}^{2}/{\left(2\sigma \right)}^{2}\right]}$$

### 3.5. Cell Culture and Proliferation Assay

Cancer cell lines, purchased from ATCC (Manassas, VA, USA), were cultured in media supplemented with 10% FBS and 1% penicillin/streptomycin (Life Technologies, Carlsbad, CA, USA). DMEM media was used for culturing A549, Mia-paca2, ASPC1, HepG2, HeLa, U87, Cal62, CHL-1, and SK-Mel28. H1299 was cultured in RPMI1640; HCT116 was cultured in McCoy’s 5A; and PC3 was cultured in F12K. For the proliferation assay, cells were seeded at a density of 5000 per well in 96-well plates in culture media containing 0.5% FBS. After incubating overnight, cells were treated with **1** at a concentration of 20 μg/mL for 48 h. Cell viability was measured using a multifunctional microplate reader (Tecan, Männedorf, Switzerland) after the addition of 100 μL Cell Titer Glo reagent (Promega, Madison, WI, USA). To determine GI_50_ values, cells were treated with various doses of **1** for 48 h before measuring cell viability. Live cell growth monitoring in real time was conducted using the IncuCyte ZOOMTM live cell imaging system (Essen BioScience, Ann Arbor, MI, USA). Briefly, 2500 cells per well were seeded in 96-well plates and cell growth after treatment with **1** was monitored for four days. Cell growth was measured by cell confluency using the IncuCyte ZOOMTM program.

### 3.6. Focus Formation Assay

A549 cells were seeded at a density of 500 per well in 6-well plates and cultured for three days. Then, cells were treated with **1** by exchange culture media containing 10 μg/mL of **1** every three days for seven days. Foci were fixed with cold methanol for 10 min, and stained with 0.5% crystal violet (Sigma-Aldrich, St. Louis, MO, USA) for 5 min at room temperature. After staining, the crystal violet solution was removed, washed with distilled water twice, and finally foci were visualized.

### 3.7. Immunofluorescence Staining

Non-small cell lung carcinoma cell lines A549 and H1299 were seeded at a density of 4 × 10^4^ cells per well in 24-well plates and treated with **1** for 24 h. For immunostaining, cells were fixed with 4% paraformaldehyde (Sigma-Aldrich, St. Louis, MO, USA) for 10 min followed by permeabilization with 0.1% triton X-100 (Sigma-Aldrich, St. Louis, MO, USA) for 10 min. Permeabilized cells were blocked with PBS containing 10% normal horse serum for 1 h at room temperature and incubated with an anti-Ki-67 antibody (Abcam, Cambridge, MA, USA) for 1 h. Then, cells were incubated with a cy3-conjugated secondary antibody to detect the primary anti-Ki-67 antibody, and 4′,6-diamidino-2-phenylindole (DAPI, Sigma-Aldrich, St. Louis, MO, USA) was added to give a concentration of 5 M for 10 min for nuclear staining. Fluorescence images were taken using a fluorescence microscope (Zeiss Axio Observer A1, Oberkochen, Germany).

### 3.8. Cell Cycle Analysis

First, 1 × 10^6^ cells were cultured in a 100-mm dish overnight, followed by treatment with **1** at 10 μg/mL for 24 h. Then, cells were trypsinized and washed with PBS three times. Cells were fixed with 4% paraformaldehyde and 50 μL of 100 μg/mL Rnase I (Sigma-Aldrich, St. Louis, MO, USA) was added to fixed cells and incubated for 1 h at 37 °C. Lastly, 200 μL of 50 μg/mL propidium iodide was added and the DNA contents of the cells were analyzed using flow cytometry (Gallios, Beckman Coulter, Brea, CA, USA).

### 3.9. Western Blot Analysis

Antibodies of CDK2, CDK4, CDK6, cyclin D1, and cyclin D3 were purchased from Cell Signaling Technologies (Danvers, MA, USA), and GAPDH antibody was purchased from Santa Cruz Biotechnology (Dallas, TX, USA). Cells were treated with **1** or DMSO for 24 h, then lysed with RIPA (radioimmunoprecipitation assay) buffer (Roche, Basel, Switzerland) containing protease and phosphatase inhibitors (Roche, Basel, Switzerland). Protein concentrations extracted from cell lysates were measured using BCA reagents (Pierce Biotechnology, Waltham, MA, USA) and 30 μg of the proteins were electrophoresed in 15% sodium dodecyl sulfate polyacrylamide gel and transferred to polyvinylidene fluoride (PVDF) membranes (EDM Millipore, Burlington, MA, USA). The membranes were blocked with 5% skim milk for 1 h, and incubated with primary antibodies against CDK2, CDK4, CDK6, cyclin D1, cyclin D3, and GAPDH at 4 °C overnight. Primary antibodies were detected by incubation with a horseradish peroxidase-conjugated secondary antibody (Bethyl Laboratories, Montgomery, TX, USA) for 1 h. Binding of antibodies was visualized using an ImageQuant LAS4000 imager (GE Healthcare, Chicago, IL, USA) after incubation with an enhanced chemiluminescence (ECL) substrate (Bio-Rad, Hercules, CA, USA).

## 4. Conclusions

In conclusion, a new tricyclic zizaane-type sesquiterpene with a phenyl group, antartin (**1**), was isolated from an Antarctic marine sediment-derived *Streptomyces* sp. Tricyclic sesquiterpenes are proving to be an especially fertile class of marine natural products [23]. However, the phenyl group in antartin (**1**) is unusual within this class of natural products. Antartin (**1**) showed moderate cytotoxicity against a wide range of cancer cell lines. Specifically, **1** appears to induce cell cycle arrest by downregulating cell cycle check proteins.

## Figures and Tables

**Table 1:** NMR spectroscopic data for antartin (**1**) ^1^ in methanol-*d*_4_.

Position	δc, Type ^2^	δ_H_, (*J* in Hz)	COSY	HMBC
1	54.2, qC			
2	38.7, CH	2.07, m	3a, 12	1, 3, 10, 11, 12
3a	41.7, CH_2_	1.49, m	2, 4	2, 12
3b		1.83, m		4, 5
4	53.9, CH	4.24, d (6.1)	3a	1, 2, 5, 6, 1′
5	146.8, qC			
6	136.4, qC			
7	42.4, qC			
8	49.1, CH	1.90, m	9a, 9b, 10, 11	1, 6
9a	25.8, CH_2_	1.67, m	8, 10a	1, 8, 11
9b		1.84, m	8, 10b	
10a	30.8, CH_2_	1.28, br	9a	
10b		1.48, m	9b	1, 5, 8
11	38.6, CH_2_	1.60, d (3.0)	8	1, 5, 7, 8, 9, 10
12	14.3, CH_3_	0.94, d (6.8)	2	1, 2, 3
13	14.0, CH_3_	1.52, s		5, 6, 7
14	25.7, CH_3_	1.04, s		6, 7, 8, 15
15	29.6, CH_3_	1.10, s		6, 7, 8, 14
1′	152.1, qC			
2′	111.9, qC			
3′	133.7, CH	7.85, dd (6.9, 2.0)	4′	1′, 5′, 6′, 7′
4′	115.3, CH	6.51, dd (6.9, 6.9)	3′, 5′	2′, 3′, 5′, 6′
5′	135.5, CH	7.31, dd (6.9, 6.9)	4′, 6′	1′, 3′, 6′
6′	113.0, CH	6.69, dd (6.9, 2.0)	5′	2′, 4′, 7′
7′	172.5, qC			

^1^ 500 MHz for ^1^H NMR and 125 MHz for ^13^C NMR. ^2^ Multiplicity was determined by the analysis of 2D NMR spectroscopic data.

**Table 2:** The effect of antartin (**1**) on cancer cell proliferation ^1^.

	**Lung (NSCLC** ^2^ **)**		**Colon**	**Prostate**	**Pancreatic**	
	**H1299**	**A549**	**HCT116**	**PC3**	**Mia-paca2**	**ASPC1**
DMSO	100 ± 4	100 ± 6.4	100 ± 1.6	100 ± 3.8	100 ± 3.8	100 ± 3.5
**1**	24.8 ± 4.5	12.2 ± 0.8	1.6 ± 0.4	13.9 ± 4.1	14.2 ± 4	63.2 ± 4.9
	**Liver**	**Cervix**	**Brain**	**Thyroid**	**Skin**	
	**HepG2**	**HeLa**	**U87**	**Cal62**	**CHL-1**	**SK-Mel28**
DMSO	100 ± 5.1	100 ± 12.9	100 ± 4.0	100 ± 2.6	100 ± 2.6	100 ± 2.4
**1**	20.5 ± 5.1	4.1 ± 3.6	0.8 ± 1.4	0.2 ± 0.0	0.4 ± 0.1	14.1 ± 2.8

^1^ Cell proliferation is shown as % of remaining cells after treatment of **1** at 20 μg/mL for 48 h compared with DMSO-treated cells, which is designated as % of viable cells ± STDEV. ^2^ Non-small-cell lung carcinoma.

## Supplementary Materials

The following are available online at http://www.mdpi.com/1660-3397/16/4/130/s1, Figure S1: Calculated ECD spectra of the stereo-isomers for **1**, Figure S2: ^1^H NMR spectrum (500 MHz) of antartin (**1**) in CD_3_OD, Figure S3: ^13^C NMR spectrum (125 MHz) of antartin (1) in CD_3_OD, Figure S4: COSY spectrum (500 MHz) of antartin A (**1**) in CD_3_OD, Figure S5: HSQC spectrum (500 MHz) of antartin A (**1**) in CD_3_OD, Figure S6: HMBC spectrum (500 MHz) of antartin A (**1**) in CD_3_OD, Figure S7: NOESY spectrum (500 MHz) of antartin A (**1**) in CD_3_OD, Figure S8: GI_50_ values for antartin (**1**), Table S1: ECD calculation of isomer A1 (1*R*, 2*R*, 4*S*, 8*S*) for antartin (**1**), Table S2: ECD calculation of isomer A2 (1*S*, 2*S*, 4*S*, 8*R*) for antartin (**1**), Table S3: ECD calculation of isomer A3 (1*R*, 2*S*, 4*S*, 8*S*) for antartin (**1**), Table S4: ECD calculation of isomer A4 (1*S*, 2*R*, 4*S*, 8*R*) for antartin (**1**), Table S5: ECD calculation of isomer B1 (1*R*, 2*R*, 4*R*, 8*S*) for antartin (**1**), Table S6: ECD calculation of isomer B2 (1*S*, 2*S*, 4*R*, 8*R*) for antartin (**1**), Table S7: ECD calculation of isomer B3 (1*R*, 2*S*, 4*R*, 8*S*) for antartin (**1**), Table S8: ECD calculation of isomer B4 (1*S*, 2*R*, 4*R*, 8*R*) for antartin (**1**).
