# Supplementary material for: Antartin, a Cytotoxic Zizaane-Type Sesquiterpenoid from a Streptomyces sp. Isolated from an Antarctic Marine Sediment
Source: Mar Drugs. 2018 Apr 16;16(4):130. doi: 10.3390/md16040130 (PMC5923417; doi:10.3390/md16040130)
Supplement: Supplementary file 1 [file marinedrugs-16-00130-s001.pdf]

Supporting Information for

Article

Antartin, a Cytotoxic Zizaane-Type Sesquiterpenoid from a *Streptomyces* sp. Isolated from an Antarctic Marine Sediment

Dayoung Kim [1,†], Eun Ju Lee [2,†], Jihye Lee [1,†], Alain S. Leutou [1], Yern-Hyerk Shin [3], Bomi Choi [1], Ji Sun Hwang [2], Dongyup Hahn [4,5], Hyukjae Choi [6], Jungwook Chin [2], Sung Jin Cho [2], Yong Deog Hong [7], Jaeyoung Ko [7], Chi Nam Seong [8], Katherine N. Maloney [9], Dong-Chan Oh [3], Inho Yang [10,*], Hayoung Hwang [2,*], and Sang-Jip Nam [1,*]

[1] Department of Chemistry and Nano Science, Ewha Womans University, Seoul 03760, Korea
[2] New Drug Development Center, Daegu-Gyeongbuk Medical Innovation Foundation (DGMIF), Daegu 41061, Korea
[3] Natural Products Research Institute, College of Pharmacy, Seoul National University, San 56-1, Sillim, Gwanak, Seoul 08826, Korea
[4] School of Food Science and Biotechnology, College of Agriculture and Life Sciences, Kyungpook National University, Daegu 41566, Korea
[5] Institute of Agricultural Science & Technology, Kyungpook National University, Daegu 41566, Korea
[6] College of Pharmacy, Yeungnam University, Gyeongsan-si, Gyeongsangbukdo 38541, Korea
[7] Materials Lab Amorepacific R&D Unit, Yongin, Gyeonggi-do 17074, Korea
[8] Department of Biology, College of Life Science and Natural Resource, Sunchon National University, Suncheon, 57922, Korea
[9] Department of Chemistry, Point Loma Nazarene University, 3900 Lomaland Drive, San Diego, CA 92106, USA
[10] Department of Convergence Study on the Ocean Science and Technology, Korea Maritime and Ocean University, Busan 49112, Korea
* Correspondence: ihyang@kmou.ac.kr (I.Y.); hwanghy@dgmif.re.kr (H.H.); sjnam@ewah.ac.kr (S.-J.N.); Tel.: +82-51-410-5398 (I.Y.); +82-53-790-5208 (H.H.); +82-2-3277-6805 (S.-J.N.)
† These authors contributed equally to this work

Table of Contents

Figure S1. Calculated ECD spectra of the stereo-isomers for **1** S1

Figure S2. ^{1}H NMR spectrum (500 MHz) of antartin (**1**) in CD_3OD S3

Figure S3. ^{13}C NMR spectrum (125 MHz) of antartin (**1**) in CD_3OD S4

Figure S4. COSY spectrum (500 MHz) of antartin A (**1**) in CD_3OD S5

Figure S5. HSQC spectrum (500 MHz) of antartin A (**1**) in CD_3OD S6

Figure S6. HMBC spectrum (500 MHz) of antartin A (**1**) in CD_3OD S7

Figure S7. NOESY spectrum (500 MHz) of antartin A (**1**) in CD_3OD S8

Figure S8. GI_{50} values for antartin (**1**) S9

Table S1. ECD calculation of isomer A1 (1*R*, 2*R*, 4*S*, 8*S*) for antartin (**1**) S11

Table S2. ECD calculation of isomer A2 (1*S*, 2*S*, 4*S*, 8*R*) for antartin (**1**) S12

Table S3. ECD calculation of isomer A3 (1*R*, 2*S*, 4*S*, 8*S*) for antartin (**1**) S13

Table S4. ECD calculation of isomer A4 (1*S*, 2*R*, 4*S*, 8*R*) for antartin (**1**) S14

Table S5. ECD calculation of isomer B1 (1*R*, 2*R*, 4*R*, 8*S*) for antartin (**1**) S15

Table S6. ECD calculation of isomer B2 (1*S*, 2*S*, 4*R*, 8*R*) for antartin (**1**) S16

Table S7. ECD calculation of isomer B3 (1*R*, 2*S*, 4*R*, 8*S*) for antartin (**1**) S17

Table S8. ECD calculation of isomer B4 (1*S*, 2*R*, 4*R*, 8*R*) for antartin (**1**) S18

Figure S1. Calculated ECD spectra of the stereo-isomers for **1**

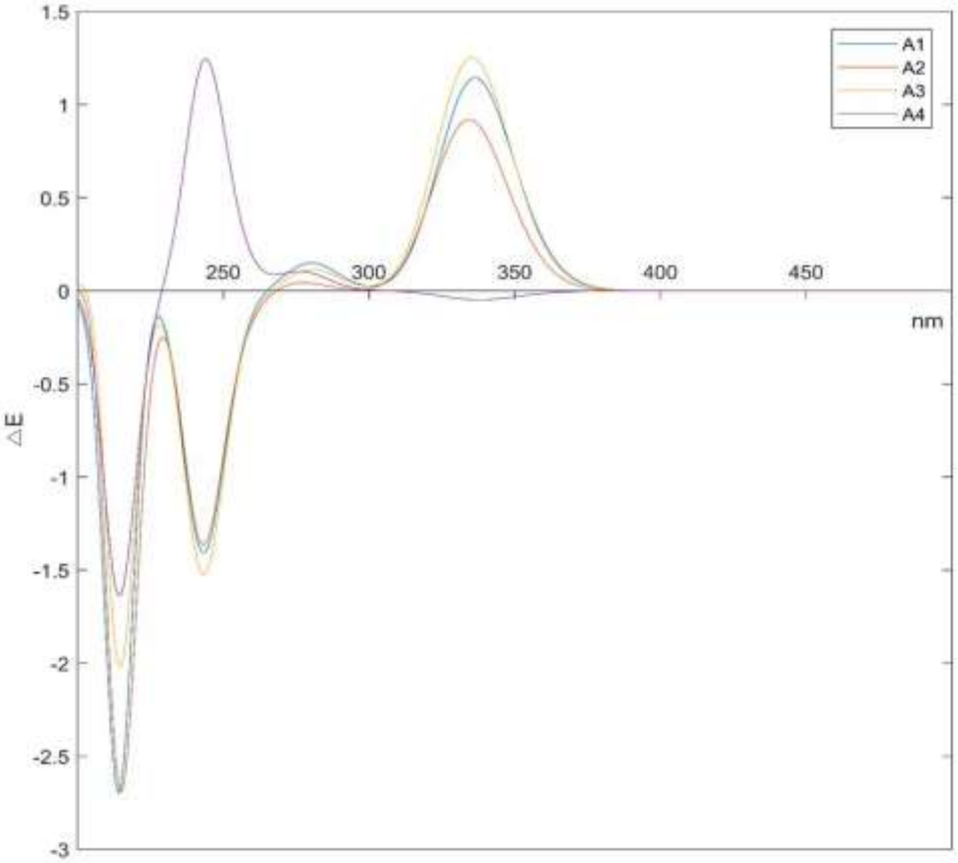

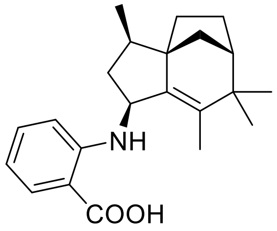

A1 (1*R*, 2*R*, 4*S*, 8*S*)

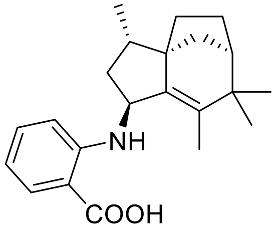

A2 (1*S*, 2*S*, 4*S*, 8*R*)

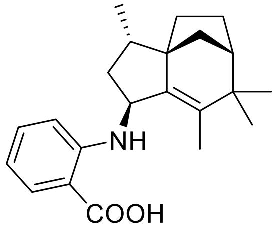

A3 (1*R*, 2*S*, 4*S*, 8*S*)

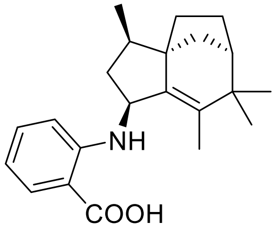

A4 (1*S*, 2*R*, 4*S*, 8*R*)

Figure S1. Calculated ECD spectra of the stereo-isomers for **1** (*Continued*)

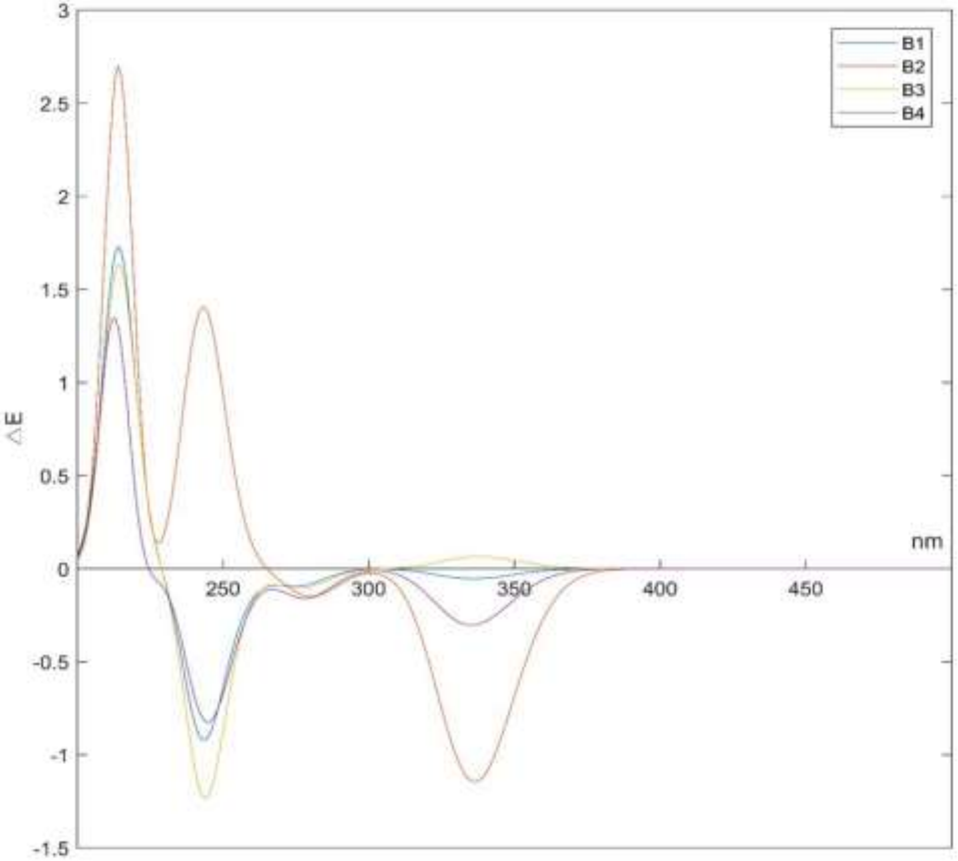

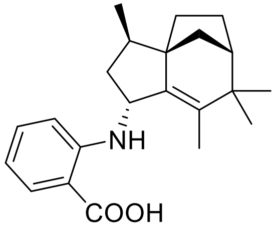

B1 (1*R*, 2*R*, 4*R*, 8*S*)

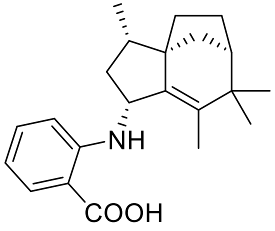

B2 (1*S*, 2*S*, 4*R*, 8*R*)

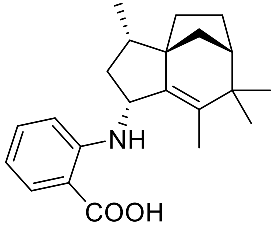

B3 **1** (1*R*, 2*S*, 4*R*, 8*S*)

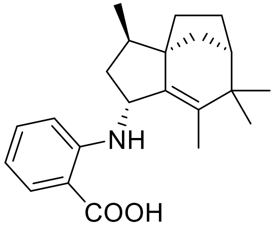

B4 (1*S*, 2*R*, 4*R*, 8*R*)

Figure S2. ^{1}H NMR spectrum (500 MHz) of antartin (**1**) in CD_3OD

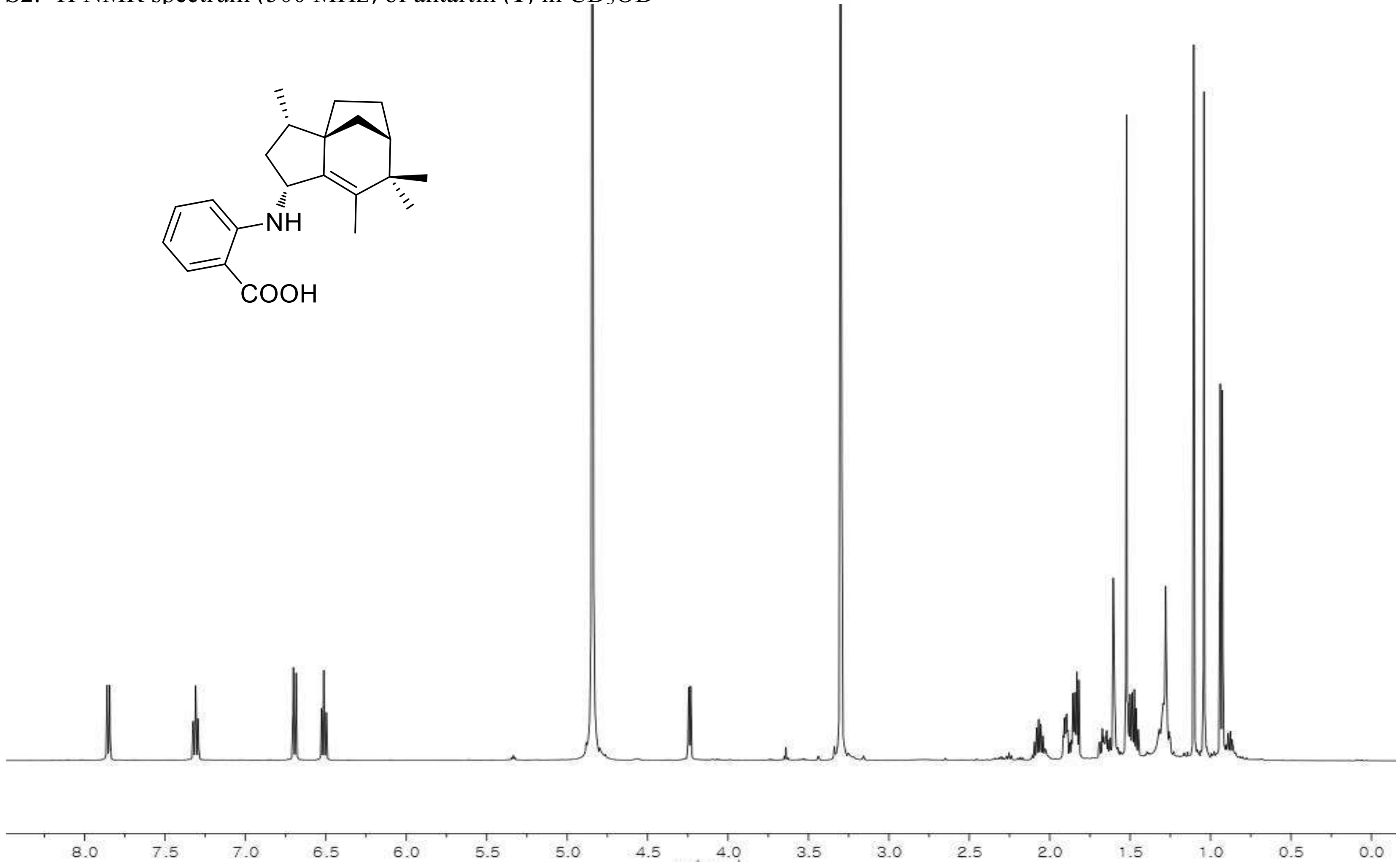

Figure S3. ^{13}C NMR spectrum (125 MHz) of antartin (**1**) in CD_3OD

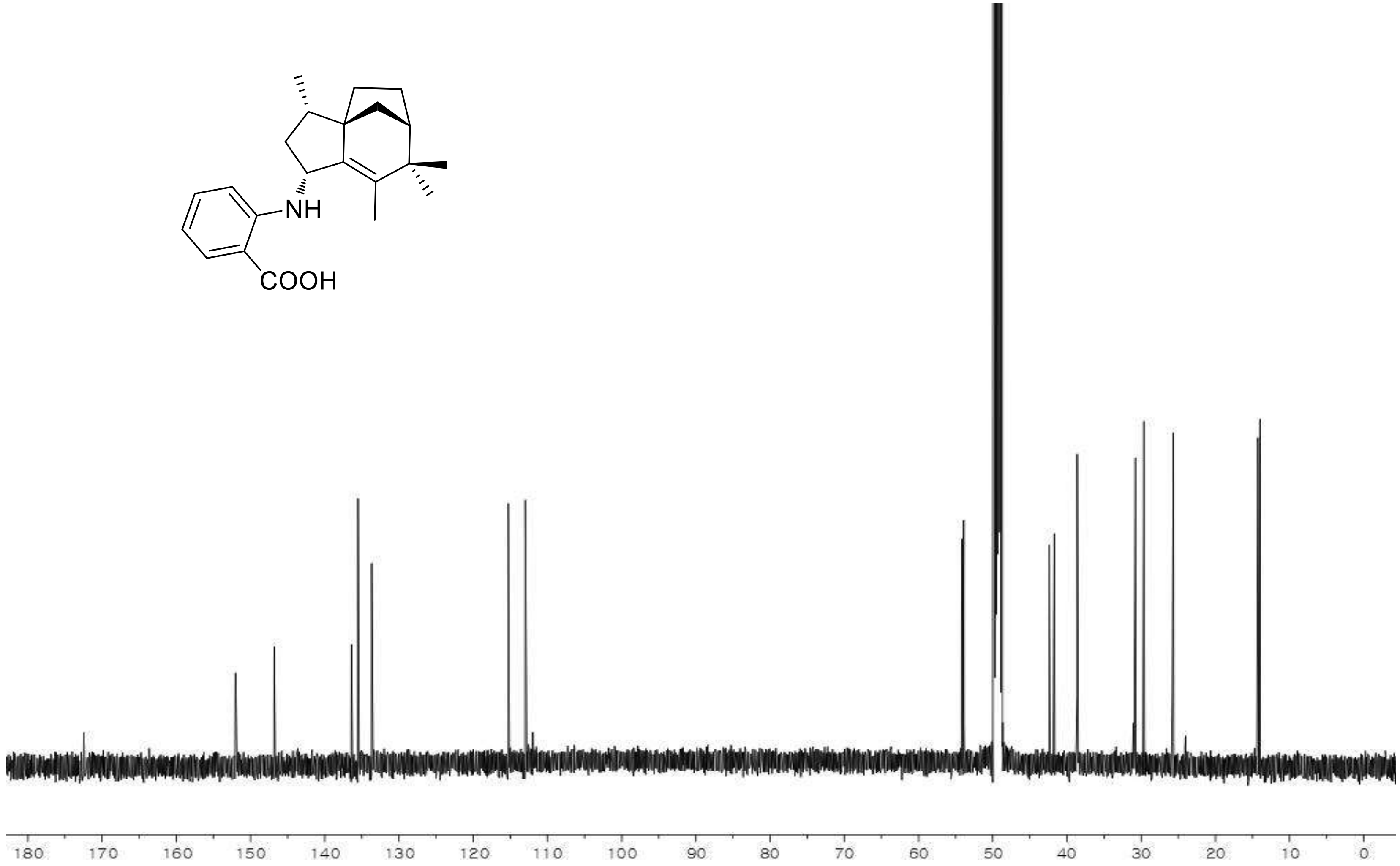

Figure S4. COSY spectrum (500 MHz) of antartin (**1**) in CD_3OD

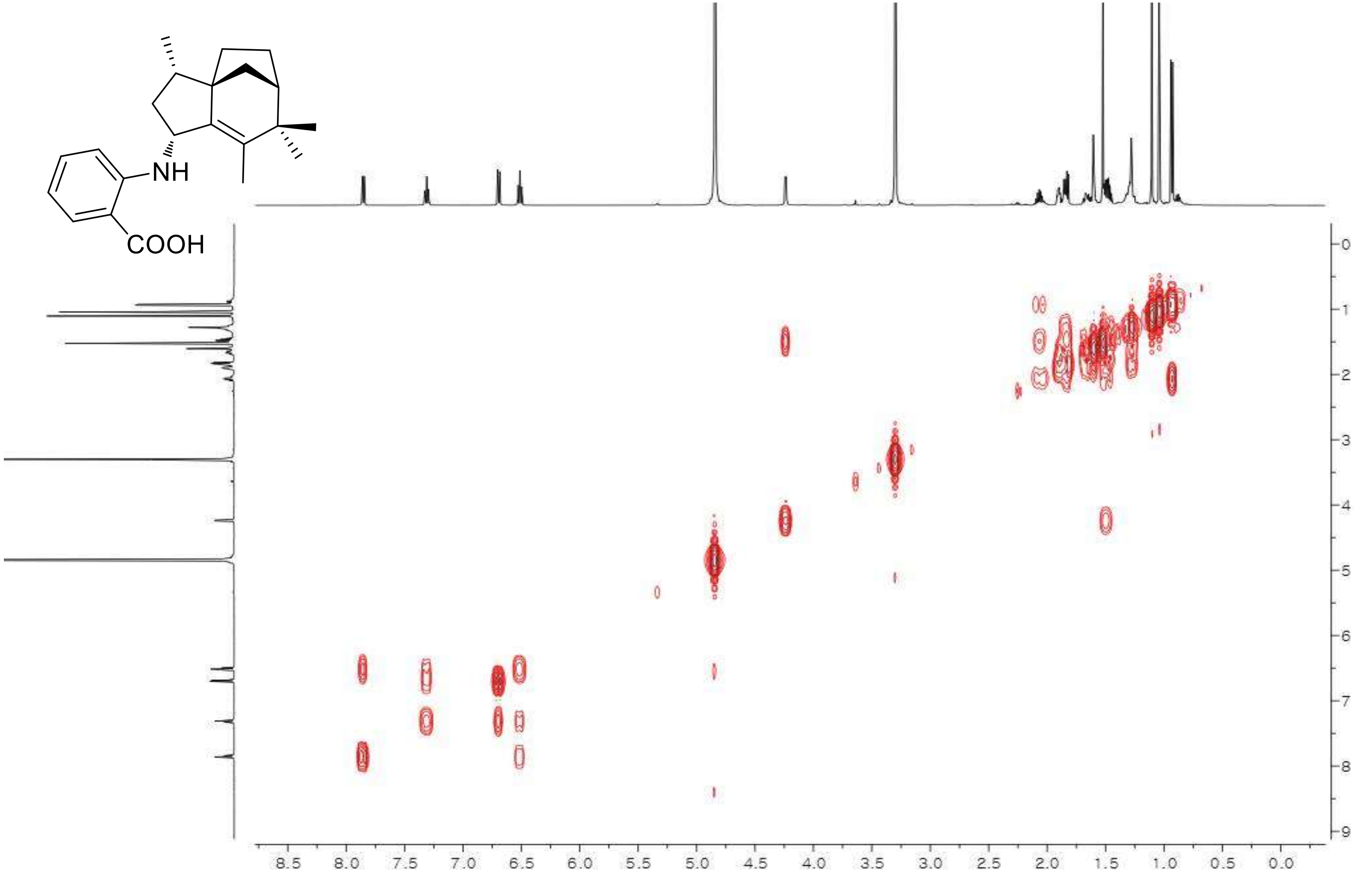

Figure S5. HSQC spectrum (500 MHz) of antartin (**1**) in CD_3OD

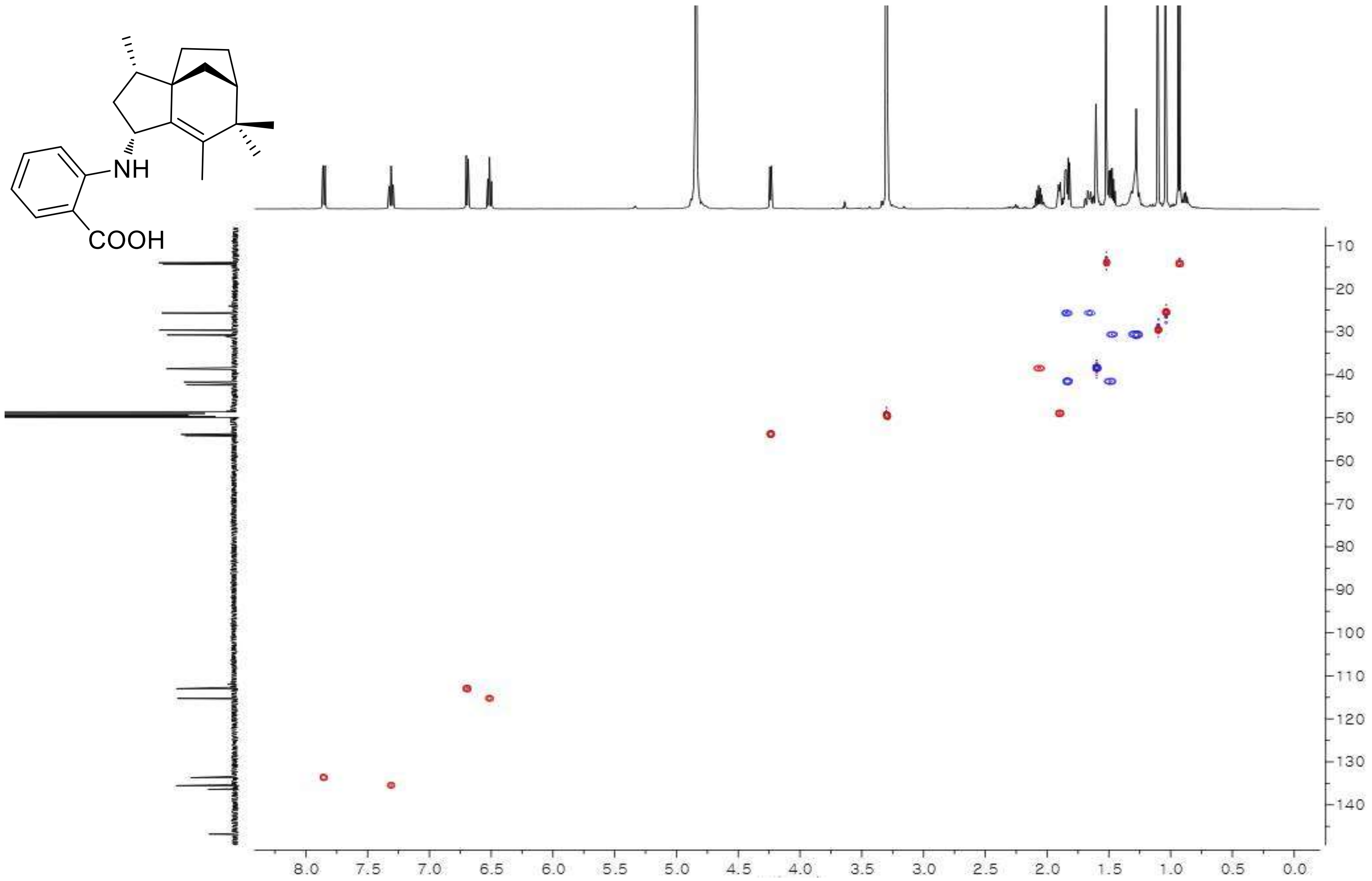

Figure S6. HMBC spectrum (500 MHz) of antartin (**1**) in CD_3OD

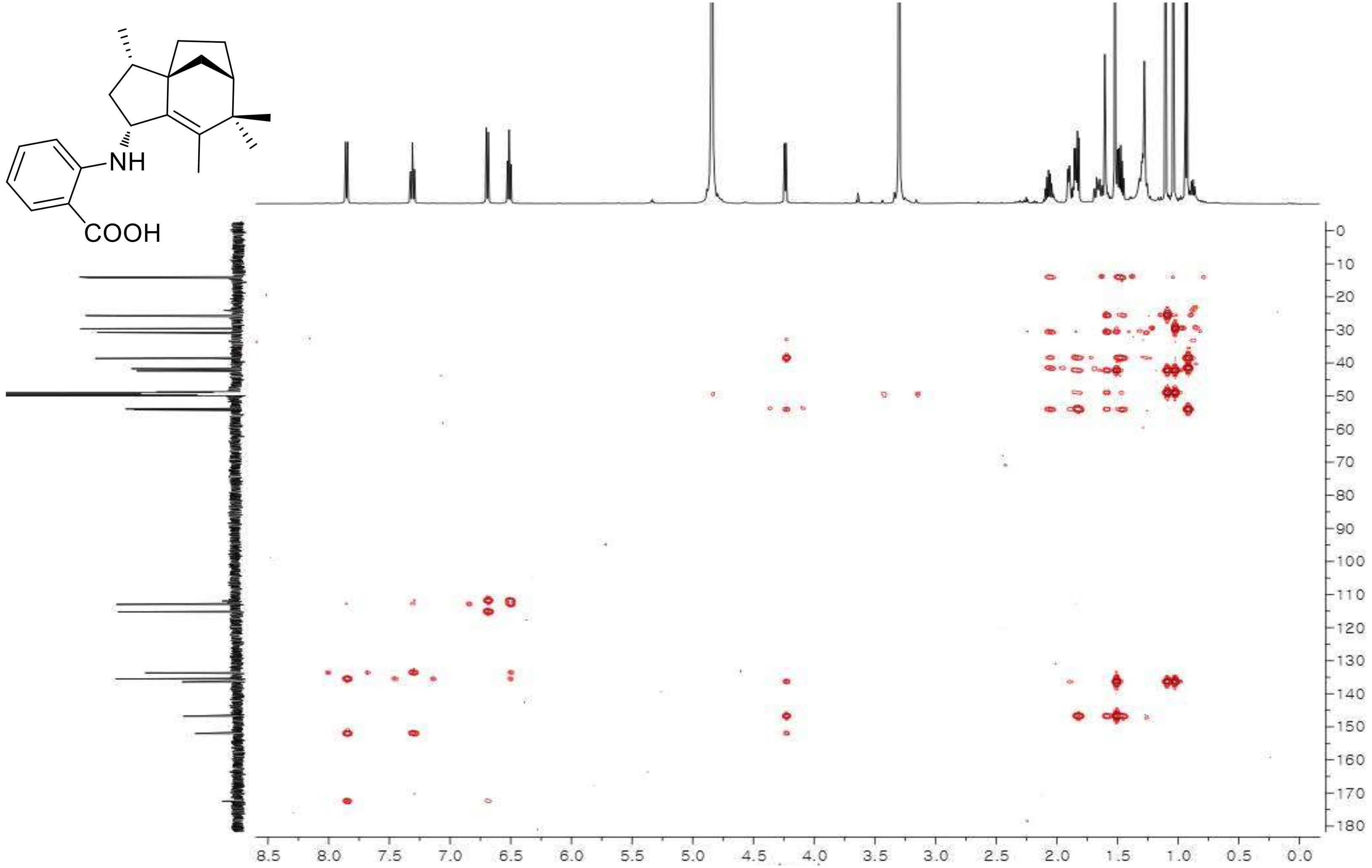

Figure S7. NOESY spectrum (500 MHz) of antartin (**1**) in CD_3OD

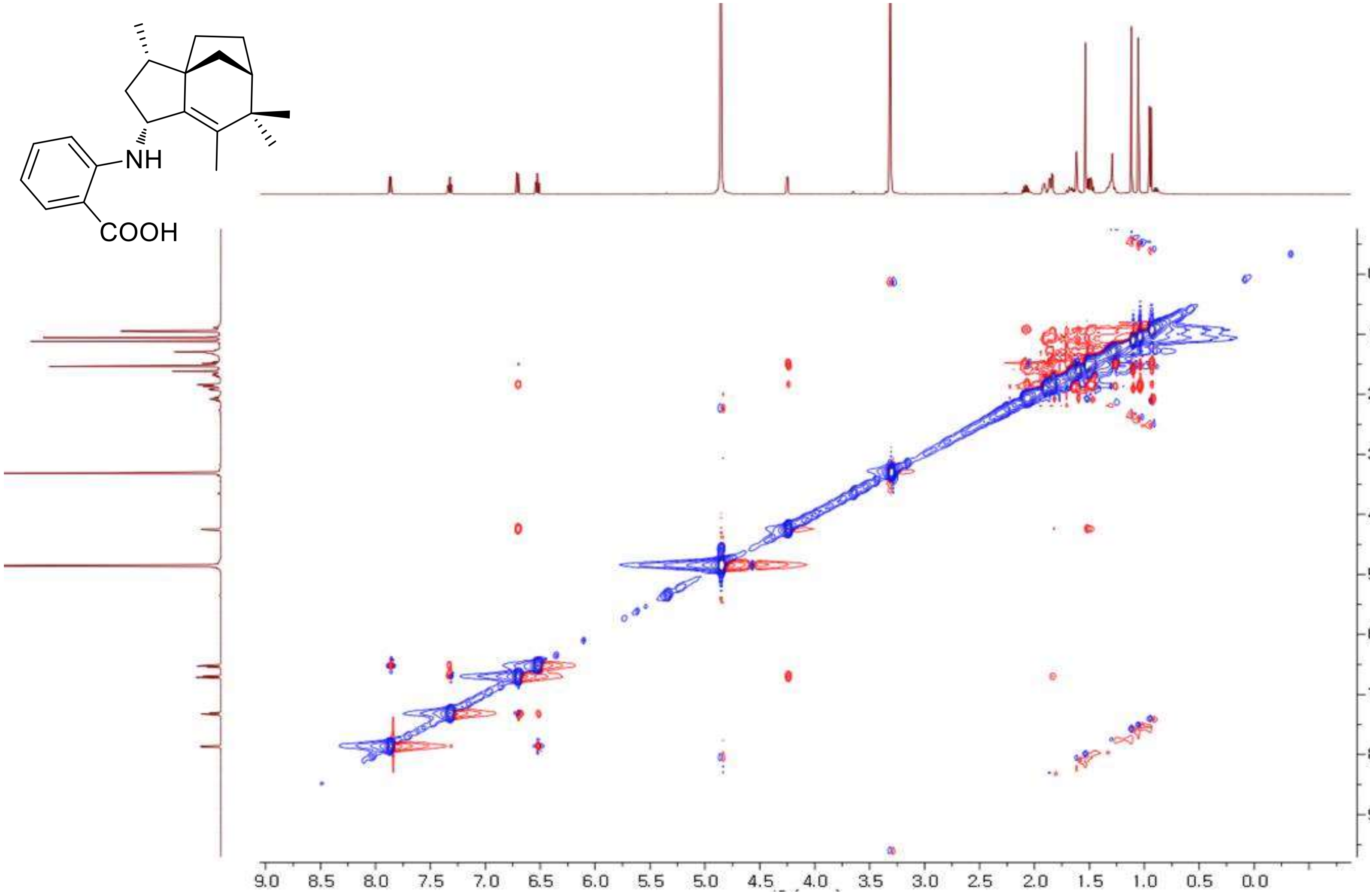

Figure S8. GI_{50} values for antartin (**1**)

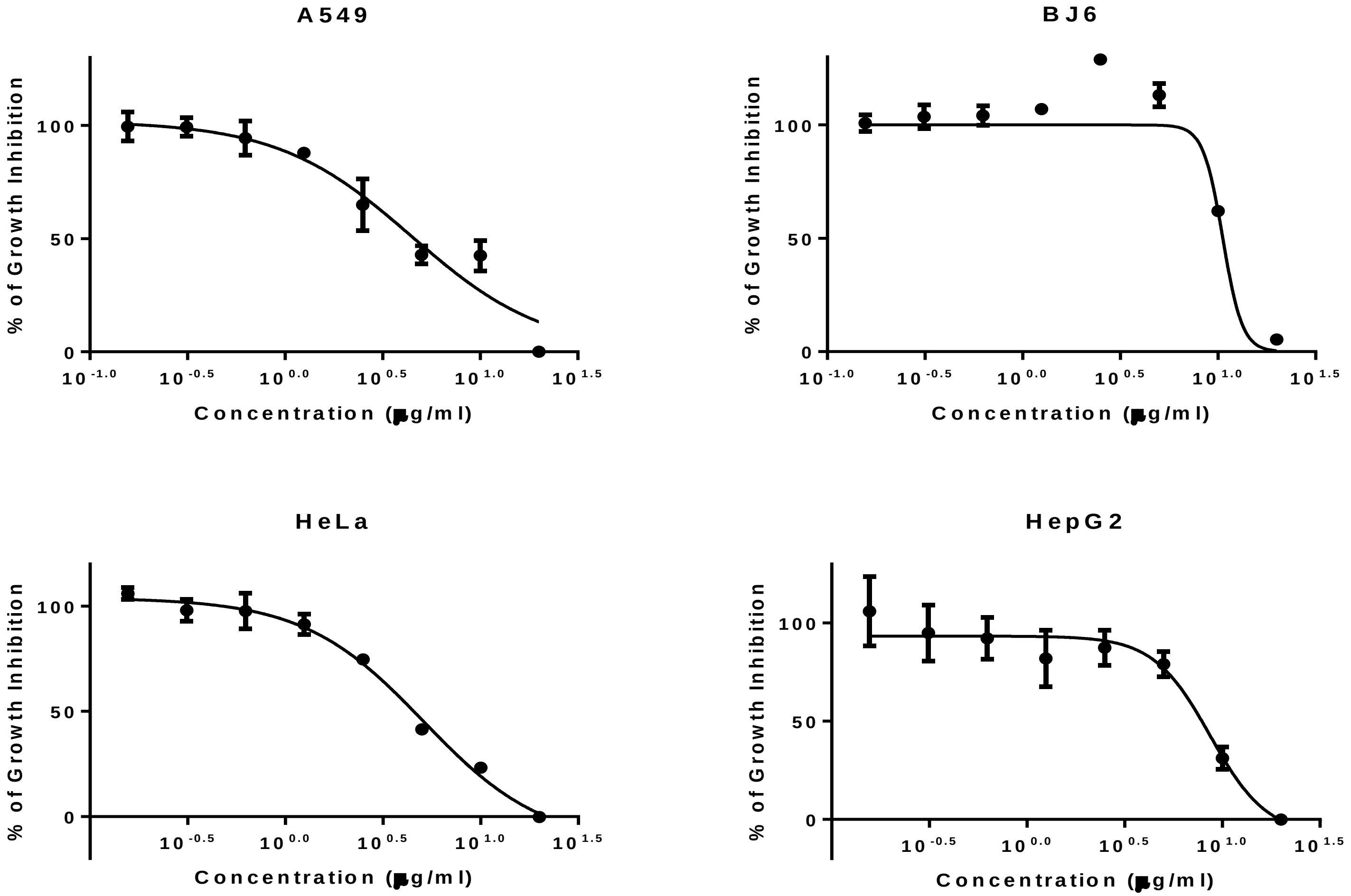

Figure S8. GI_{50} values for antartin (**1**) (*continued*)

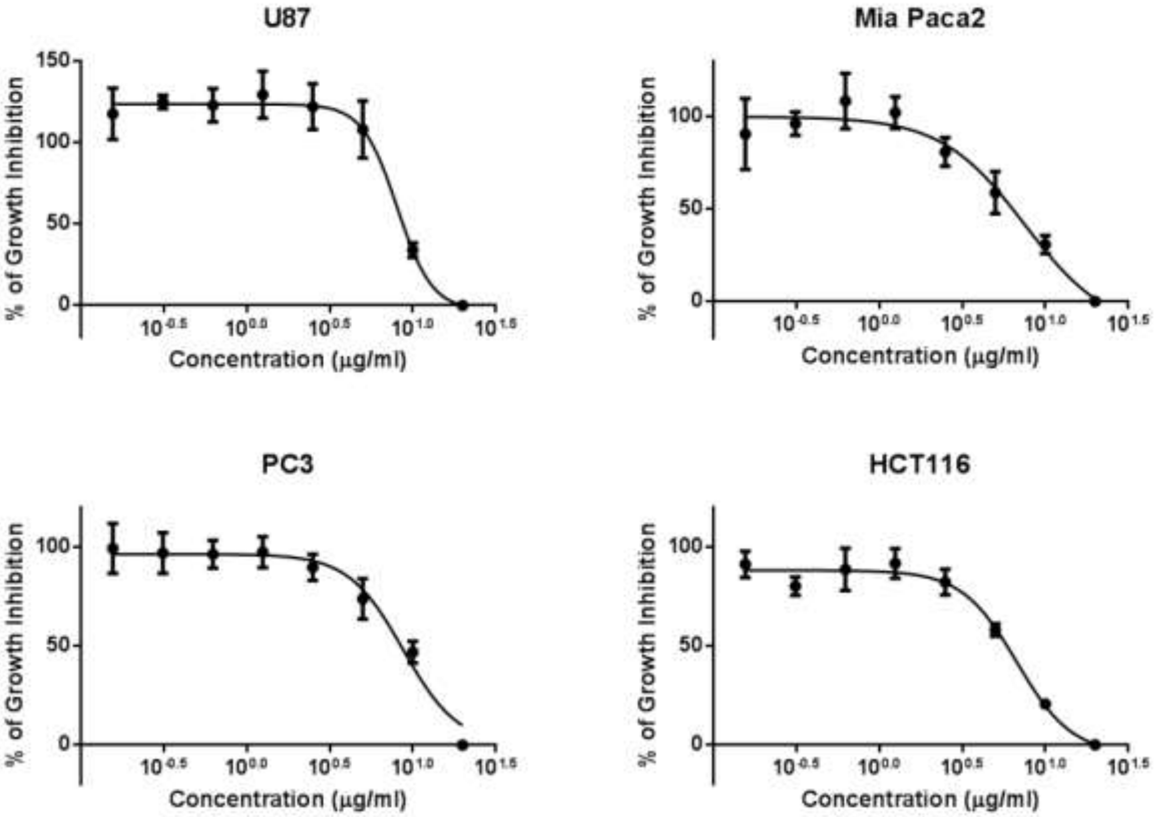

Cell line	A549	HeLa	HepG2	U87	Mia-paca2	PC3	HCT116	BJ6
GI_{50} (μg/ml)	4.45	5.09	8.66	8.06	7.54	8.75	6.75	10.59

Table S1. ECD calculation of isomer A1 (1*R*, 2*R*, 4*S*, 8*S*) for antartin (**1**)

total energy = -1059.50579029099
kinetic energy = 1048.92994898336
potential energy = -2108.43573927435

Parameters of Level DFT
DFT settings (Functional B3-LYP / Gridsize M3)
Geometry optimization options (Energy 10^{-6} Hartree, Gradient norm $|dE/dxyz|$ = 10^{-3} Hartree/Bohr)

Energy minimized coordinates of isomer 2 (1*R*, 2*R*, 4*S*, 8*S*) at the basis set def-SV(P) for all atoms (Å).

Atom	X	Y	Z	Atom	X	Y	Z
C	2.8335	-2.2456	-6.9450	H	7.5822	0.4737	-5.3536
C	3.9141	-3.9909	-4.8641	H	7.3004	4.9268	-4.5799
C	3.3268	-2.6543	-2.3147	H	6.4960	5.3349	-7.7874
H	3.1308	-5.9213	-4.9331	H	-1.5987	4.5339	-3.0517
H	5.9846	-4.1518	-5.0837	H	-0.4172	7.6931	-2.8531
C	3.1584	0.1447	-3.0301	H	-0.7041	5.8139	-0.0773
N	0.9655	-3.4724	-1.1452	H	6.2588	5.9860	-0.9195
H	4.8812	-2.9828	-0.9550	H	3.6116	7.0292	0.8686
C	3.4129	0.4288	-5.8984	H	4.3391	8.5194	-2.0546
C	0.0166	-2.7310	-7.4549	H	-0.2799	-4.7049	-8.0715
H	3.8634	-2.5436	-8.7377	H	-0.7084	-1.4841	-8.9638
C	6.1226	1.3980	-6.5196	H	-1.1547	-2.4255	-5.7564
C	1.8013	2.6830	-6.7641	H	-0.5093	-2.2323	-1.1101
C	2.8154	2.1076	-1.4415	C	0.6068	-5.6417	0.1849
C	3.1671	4.8557	-5.3869	C	2.5851	-7.4335	0.5144
H	-0.2075	2.4927	-6.2605	C	2.2454	-9.5975	1.9578
H	1.9211	2.9386	-8.8367	C	-0.0725	-10.1057	3.1433
C	2.6128	1.7656	1.3893	C	-2.0479	-8.4104	2.7967
C	2.5468	4.8138	-2.4956	C	-1.8035	-6.2102	1.3134
C	4.2972	6.6768	-1.0699	H	4.4149	-7.0962	-0.3736
C	-0.2028	5.7546	-2.1008	H	3.8266	-10.9150	2.1791
H	2.5979	6.7258	-6.1144	H	-0.3220	-11.7862	4.3185
C	5.9777	4.2964	-6.0539	H	-3.8324	-8.7876	3.7790
H	2.9361	-0.2042	1.9694	C	-3.9785	-4.4688	0.9465
H	0.7209	2.3083	2.0947	O	-3.8089	-2.3194	0.1516
H	3.9974	2.9596	2.4000	O	-6.3094	-5.3563	1.5274
H	6.5715	0.9816	-8.5166	H	-6.2624	-7.1645	1.8468

Table S2. ECD calculation of isomer A2 (1*S*, 2*S*, 4*S*, 8*R*) for antartin (**1**)

total energy = -1059.50505206597
kinetic energy = 1048.92152437166
potential energy = -2108.42657643763

Parameters of Level DFT
DFT settings (Functional B3-LYP / Gridsize M3)
Geometry optimization options (Energy 10^{-6} Hartree, Gradient norm $|dE/dxyz|$ = 10^{-3} Hartree/Bohr)

Energy minimized coordinates of isomer 8 (1*S*, 2*S*, 4*S*, 8*R*) at the basis set def-SV(P) for all atoms (Å).

Atom	X	Y	Z	Atom	X	Y	Z
C	6.9391	-2.9562	-4.2390	H	4.4312	-0.4418	-7.9362
C	6.9852	-3.8848	-1.4793	H	4.1563	3.9532	-8.2002
C	4.7365	-2.5306	-0.1894	H	1.8651	4.0570	-5.7831
H	8.7509	-3.2682	-0.5407	H	0.9068	5.6388	-2.2824
H	6.8821	-5.9625	-1.3269	H	2.9873	8.2118	-2.9516
C	4.8661	0.0741	-1.4066	H	2.3101	7.3699	0.2328
N	2.2935	-3.7059	-0.7153	H	8.8950	5.0771	-0.2256
H	5.0186	-2.4350	1.8751	H	6.6253	6.6790	1.6760
C	5.9192	-0.1899	-4.0978	H	7.5760	8.0062	-1.2613
C	9.4397	-3.3154	-5.6435	H	10.9969	-2.2671	-4.7262
H	5.4884	-4.0831	-5.2347	H	9.3118	-2.6604	-7.6228
C	3.8504	0.3822	-6.1052	H	9.9790	-5.3341	-5.6898
C	7.7417	2.0414	-4.5302	H	1.0268	-2.7426	-1.8021
C	4.3800	2.3130	-0.2902	C	1.3711	-5.8441	0.3768
C	5.8549	4.2655	-4.4521	C	2.7763	-7.2072	2.2201
H	8.6440	1.9305	-6.4113	C	1.7866	-9.3310	3.4011
H	9.2566	2.1595	-3.1041	C	-0.6440	-10.2186	2.8161
C	3.3031	2.5106	2.3503	C	-2.0270	-8.9474	0.9805
C	4.9739	4.8064	-1.6801	C	-1.0812	-6.8095	-0.2988
C	7.1443	6.2078	-0.2882	H	4.6627	-6.5583	2.7383
C	2.6575	6.5923	-1.6721	H	2.9250	-10.3086	4.8268
H	6.6990	6.0280	-5.1825	H	-1.4402	-11.8607	3.7840
C	3.7444	3.3080	-6.2595	H	-3.9477	-9.6101	0.5770
H	2.9647	0.6467	3.2084	C	-2.5951	-5.5392	-2.2976
H	1.4692	3.5121	2.3355	O	-2.1902	-3.4228	-3.0943
H	4.5560	3.5862	3.6327	O	-4.5522	-6.8509	-3.3024
H	2.0200	-0.4816	-5.6077	H	-4.5140	-8.6050	-2.7576

Table S3. ECD calculation of isomer A3 (1*R*, 2*S*, 4*S*, 8*S*) for antartin (**1**)

total energy = -1059.50707767898
kinetic energy = 1048.93306606935
potential energy = -2108.44014374833

Parameters of Level DFT
DFT settings (Functional B3-LYP / Gridsize M3)
Geometry optimization options (Energy 10^{-6} Hartree, Gradient norm $|dE/dxyz|$ = 10^{-3} Hartree/Bohr)

Energy minimized coordinates of isomer 4 (1*R*, 2*S*, 4*S*, 8*S*) at the basis set def-SV(P) for all atoms (Å).

Atom	X	Y	Z	Atom	X	Y	Z
C	3.2502	-2.1559	-7.0361	H	7.9015	0.8221	-5.3088
C	4.3459	-3.8853	-4.9672	H	7.3310	5.2492	-4.5405
C	3.5941	-2.5944	-2.4462	H	6.5868	5.6206	-7.7659
H	3.6471	-5.8453	-5.0877	H	-1.5758	4.4314	-3.2838
H	6.4300	-3.9469	-5.1290	H	-0.5115	7.6216	-2.9473
C	3.4054	0.2079	-3.1430	H	-0.8171	5.6412	-0.2419
N	1.1700	-3.4683	-1.4571	H	6.1515	6.0804	-0.8492
H	5.0598	-2.9119	-0.9877	H	3.4097	7.0869	0.8123
C	3.7659	0.5326	-5.9953	H	4.2380	8.5735	-2.0827
C	4.1716	-2.7469	-9.7103	H	6.2562	-2.7402	-9.8403
H	1.1717	-2.4054	-7.0064	H	3.4401	-1.3671	-11.0989
C	6.4301	1.6610	-6.5234	H	3.5172	-4.6384	-10.3081
C	2.0430	2.6912	-6.8909	H	-0.3077	-2.2334	-1.4753
C	2.8652	2.1254	-1.5545	C	0.7811	-5.6252	-0.1126
C	3.2326	4.9310	-5.4577	C	2.7565	-7.4036	0.2937
H	0.0368	2.3525	-6.4429	C	2.3786	-9.5673	1.7289
H	2.2019	2.9775	-8.9567	C	0.0232	-10.0874	2.8317
C	2.4257	1.6964	1.2406	C	-1.9452	-8.3975	2.4216
C	2.5365	4.8311	-2.5844	C	-1.6573	-6.1944	0.9507
C	4.1865	6.7348	-1.0911	H	4.6136	-7.0605	-0.5330
C	-0.2534	5.6677	-2.2504	H	3.9585	-10.8753	2.0080
H	2.5769	6.7716	-6.1875	H	-0.2594	-11.7692	3.9976
C	6.0881	4.5441	-6.0497	H	-3.7583	-8.7754	3.3495
H	3.2544	-0.0988	1.8926	C	-3.8159	-4.4361	0.5607
H	0.3842	1.6184	1.6876	O	-3.6162	-2.2626	-0.1558
H	3.2479	3.2257	2.3963	O	-6.1653	-5.3382	1.0347
H	6.9874	1.3079	-8.5018	H	-6.1277	-7.1562	1.2955

Table S4. ECD calculation of isomer A4 (1*S*, 2*R*, 4*S*, 8*R*) for antartin (**1**)

total energy = -1059.50129871075
kinetic energy = 1048.91445929629
potential energy = -2108.41575800704

Parameters of Level DFT
DFT settings (Functional B3-LYP / Gridsize M3)
Geometry optimization options (Energy 10^{-6} Hartree, Gradient norm $|dE/dxyz|$ = 10^{-3} Hartree/Bohr)

Energy minimized coordinates of isomer 6 (1*S*, 2*R*, 4*S*, 8*R*) at the basis set def-SV(P) for all atoms (Å).

Atom	X	Y	Z	Atom	X	Y	Z
C	7.7700	-2.1973	-3.9014	H	4.9381	-0.0217	-7.8513
C	7.4420	-3.2267	-1.1696	H	4.0747	4.2723	-8.1239
C	4.9977	-2.0213	-0.1086	H	1.8298	4.1076	-5.6697
H	9.0359	-2.6047	0.0283	H	0.7269	5.9051	-2.3749
H	7.3949	-5.3104	-1.1007	H	2.6715	8.6035	-2.9443
C	5.0578	0.5821	-1.3205	H	1.9402	7.6799	0.2063
N	2.6919	-3.3614	-0.8285	H	8.6774	5.8311	-0.0937
H	5.0875	-1.9048	1.9716	H	6.2661	7.3011	1.7354
C	6.2744	0.3653	-3.9471	H	7.2063	8.6644	-1.1892
C	6.9949	-4.1504	-5.9053	H	7.3345	-3.4587	-7.8442
H	9.7902	-1.7730	-4.2128	H	4.9786	-4.6641	-5.7506
C	4.2177	0.6885	-6.0304	H	8.1153	-5.8978	-5.6692
C	7.8614	2.7637	-4.3903	H	1.4732	-2.5127	-2.0559
C	4.3408	2.7867	-0.2595	C	1.8353	-5.5578	0.1979
C	5.7776	4.7979	-4.3912	C	3.1544	-6.7924	2.1903
H	8.8113	2.6834	-6.2525	C	2.2219	-8.9789	3.3008
H	9.3318	3.0373	-2.9396	C	-0.0627	-10.0605	2.4944
C	3.0721	2.9290	2.2979	C	-1.3519	-8.9174	0.5125
C	4.8170	5.3085	-1.6436	C	-0.4541	-6.7217	-0.7022
C	6.8625	6.8493	-0.2116	H	4.9248	-5.9935	2.8794
C	2.3968	6.9509	-1.6944	H	3.2871	-9.8532	4.8453
H	6.4533	6.6150	-5.1619	H	-0.8197	-11.7534	3.4042
C	3.7774	3.5887	-6.1756	H	-3.1641	-9.7366	-0.0668
H	3.0526	1.0968	3.2825	C	-1.8474	-5.5982	-2.8678
H	1.0810	3.5374	2.1069	O	-1.5015	-3.4768	-3.6817
H	3.9981	4.3171	3.5556	O	-3.6106	-7.0548	-4.0195
H	2.4718	-0.3694	-5.6209	H	-3.5213	-8.7900	-3.4234

Table S5. ECD calculation of isomer B1 (1*R*, 2*R*, 4*R*, 8*S*) for antartin (**1**)

total energy = -1059.50459137516
kinetic energy = 1048.92529189881
potential energy = -2108.42988327397

Parameters of Level DFT
DFT settings (Functional B3-LYP / Gridsize M3)
Geometry optimization options (Energy 10^{-6} Hartree, Gradient norm $|dE/dxyz|$ = 10^{-3} Hartree/Bohr)

Energy minimized coordinates of isomer 1 (1*R*, 2*R*, 4*R*, 8*S*) at the basis set def-SV(P) for all atoms (Å).

Atom	X	Y	Z	Atom	X	Y	Z
C	7.7925	-2.4107	-0.9989	H	9.5559	2.0583	2.0400
C	5.1029	-3.2883	-0.3133	H	9.4587	6.1636	0.0880
C	3.9839	-1.0924	1.2652	H	11.5636	5.0188	-2.2265
H	5.0809	-5.1117	0.6984	H	3.0061	3.8400	-5.7662
H	3.9519	-3.5092	-2.0472	H	3.9876	7.0572	-6.0527
C	5.0146	1.1988	-0.1430	H	1.2894	6.2808	-4.2059
N	4.8344	-1.0724	3.8908	H	5.9869	7.9537	0.7548
H	1.9007	-1.1440	1.2331	H	3.2063	8.8544	-0.9049
C	7.5772	0.5143	-1.3209	H	6.2187	9.3171	-2.3249
C	8.9815	-3.8480	-3.2061	H	9.1077	-5.8891	-2.7763
H	8.9854	-2.7424	0.6847	H	7.8600	-3.6388	-4.9558
C	9.7712	1.9850	-0.0318	H	10.9188	-3.1767	-3.6082
C	7.6239	1.6732	-3.9936	H	6.2371	0.1467	4.3973
C	3.8132	3.4069	-0.5582	C	4.0113	-2.6177	5.7719
C	7.6190	4.5119	-3.3193	C	2.1131	-4.4737	5.3378
H	6.0191	1.0768	-5.1802	C	1.3017	-6.0777	7.2474
H	9.3848	1.1802	-5.0043	C	2.3349	-5.9363	9.6873
C	1.3047	4.0384	0.6579	C	4.1593	-4.1089	10.1635
C	4.9466	5.3753	-2.3866	C	5.0069	-2.4049	8.2967
C	5.1025	8.0137	-1.1322	H	1.2949	-4.6592	3.4550
C	3.2034	5.6398	-4.7323	H	-0.1514	-7.4892	6.8230
H	8.1101	5.7029	-4.9610	H	1.7371	-7.2386	11.1750
C	9.7398	4.6337	-1.2899	H	4.9975	-4.0547	12.0572
H	0.6593	2.5488	1.9585	C	6.9228	-0.4362	8.8954
H	-0.2017	4.3485	-0.7582	O	8.0616	0.7952	7.3248
H	1.4384	5.7991	1.7752	O	7.4157	0.0226	11.3658
H	11.5722	0.9964	-0.4141	H	6.2114	-0.8414	12.4502

Table S6. ECD calculation of isomer B2 (1*S*, 2*S*, 4*R*, 8*R*) for antartin (**1**)

total energy = -1059.50579029097
kinetic energy = 1048.92994888924
potential energy = -2108.43573918021

Parameters of Level DFT
DFT settings (Functional B3-LYP / Gridsize M3)
Geometry optimization options (Energy 10^{-6} Hartree, Gradient norm $|dE/dxyz|$ = 10^{-3} Hartree/Bohr)

Energy minimized coordinates of isomer 7 (1*S*, 2*S*, 4*R*, 8*R*) at the basis set def-SV(P) for all atoms (Å).

Atom	X	Y	Z	Atom	X	Y	Z
C	-2.8335	-2.2456	-6.9450	H	-7.5822	0.4737	-5.3536
C	-3.9141	-3.9909	-4.8641	H	-7.3004	4.9268	-4.5799
C	-3.3268	-2.6543	-2.3147	H	-6.4960	5.3349	-7.7874
H	-3.1308	-5.9213	-4.9331	H	1.5987	4.5339	-3.0517
H	-5.9846	-4.1518	-5.0837	H	0.4172	7.6931	-2.8531
C	-3.1584	0.1447	-3.0301	H	0.7041	5.8139	-0.0773
N	-0.9655	-3.4724	-1.1452	H	-6.2588	5.9860	-0.9195
H	-4.8812	-2.9828	-0.9550	H	-3.6116	7.0292	0.8686
C	-3.4129	0.4288	-5.8984	H	-4.3391	8.5195	-2.0546
C	-0.0166	-2.7310	-7.4549	H	0.2799	-4.7049	-8.0715
H	-3.8634	-2.5436	-8.7377	H	0.7084	-1.4841	-8.9638
C	-6.1226	1.3980	-6.5196	H	1.1547	-2.4255	-5.7564
C	-1.8013	2.6830	-6.7641	H	0.5093	-2.2323	-1.1101
C	-2.8154	2.1076	-1.4415	C	-0.6068	-5.6417	0.1849
C	-3.1671	4.8557	-5.3869	C	-2.5851	-7.4335	0.5144
H	0.2075	2.4927	-6.2605	C	-2.2454	-9.5975	1.9578
H	-1.9211	2.9386	-8.8367	C	0.0725	-10.1057	3.1433
C	-2.6128	1.7656	1.3893	C	2.0479	-8.4104	2.7967
C	-2.5468	4.8138	-2.4956	C	1.8035	-6.2102	1.3134
C	-4.2972	6.6768	-1.0699	H	-4.4149	-7.0962	-0.3736
C	0.2028	5.7546	-2.1008	H	-3.8266	-10.9150	2.1791
H	-2.5979	6.7258	-6.1144	H	0.3220	-11.7862	4.3185
C	-5.9777	4.2964	-6.0539	H	3.8324	-8.7876	3.7790
H	-2.9361	-0.2042	1.9694	C	3.9785	-4.4688	0.9465
H	-0.7209	2.3083	2.0947	O	3.8089	-2.3194	0.1516
H	-3.9974	2.9596	2.4000	O	6.3094	-5.3563	1.5274
H	-6.5715	0.9816	-8.5166	H	6.2624	-7.1645	1.8468

Table S7. ECD calculation of isomer B3 (1*R*, 2*S*, 4*R*, 8*S*) for antartin (**1**)

total energy	=	-1059.50113948987
kinetic energy	=	1048.90962446441
potential energy	=	-2108.41076395428

Parameters of Level DFT
DFT settings (Functional B3-LYP / Gridsize M3)
Geometry optimization options (Energy 10^{-6} Hartree, Gradient norm $|dE/dxyz|$ = 10^{-3} Hartree/Bohr)
Energy minimized coordinates of antartin (**1**) at the basis set def-SV(P) for all atoms (Å).

Atom	X	Y	Z	Atom	X	Y	Z
C	5.9947	-2.6059	-3.2703	H	8.9969	1.0276	0.2308
C	3.3970	-3.1616	-2.0239	H	9.1606	5.3641	-1.0484
C	2.9805	-1.0657	-0.0257	H	10.8678	4.3844	-3.7348
H	1.8724	-3.0034	-3.4439	H	1.8446	4.7733	-6.2706
H	3.2886	-5.0770	-1.2066	H	3.2548	7.8379	-6.3777
C	4.1324	1.2134	-1.3448	H	0.7303	7.2204	-4.2456
N	4.2461	-1.5428	2.3810	H	6.2035	7.6581	0.1352
H	0.9480	-0.7960	0.3375	H	3.3820	9.0890	-0.9971
C	6.3580	0.3263	-2.9871	H	6.2146	9.3410	-2.7905
C	8.1143	-4.2529	-2.1579	H	7.6281	-6.2741	-2.3660
H	5.8735	-3.0567	-5.3048	H	9.9305	-3.9539	-3.1404
C	8.9044	1.2582	-1.8368	H	8.4172	-3.8798	-0.1283
C	6.2890	1.8674	-5.4562	H	5.9616	-0.7266	2.6957
C	3.2525	3.6057	-1.3121	C	3.4765	-3.1838	4.2036
C	6.7997	4.5458	-4.4342	C	1.1820	-4.5684	3.9735
H	4.4875	1.6654	-6.4839	C	0.4064	-6.2535	5.8278
H	7.8261	1.2680	-6.7423	C	1.8697	-6.6705	8.0019
C	1.0791	4.4200	0.3570	C	4.1037	-5.3186	8.2777
C	4.4103	5.6246	-3.0678	C	4.9450	-3.5540	6.4655
C	5.0947	8.0522	-1.5853	H	0.0161	-4.3212	2.2919
C	2.4422	6.3939	-5.1039	H	-1.3669	-7.2883	5.5657
H	7.3019	5.8847	-5.9534	H	1.2842	-8.0392	9.4340
C	9.1020	4.0669	-2.6711	H	5.2725	-5.7101	9.9429
H	0.3828	2.8754	1.5623	C	7.3360	-2.1274	6.8428
H	-0.5377	5.1417	-0.7559	O	8.4455	-0.9821	5.1873
H	1.6555	5.9698	1.6343	O	8.3357	-2.0987	9.1995
H	10.4846	0.1584	-2.6331	H	7.1605	-2.8072	10.4208

Table S8. ECD calculation of isomer B4 (1*S*, 2*R*, 4*R*, 8*R*) for antartin (**1**)

total energy = -1059.50655280786
kinetic energy = 1048.92579828570
potential energy = -2108.43235109357

Parameters of Level DFT
DFT settings (Functional B3-LYP / Gridsize M3)
Geometry optimization options (Energy 10^{-6} Hartree, Gradient norm $|dE/dxyz|$ = 10^{-3} Hartree/Bohr)

Energy minimized coordinates of isomer 5 (1*S*, 2*R*, 4*R*, 8*R*) at the basis set def-SV(P) for all atoms (Å).

Atom	X	Y	Z	Atom	X	Y	Z
C	7.8788	0.3865	-0.2984	H	7.8209	2.8977	-4.9694
C	5.5053	-1.2906	-0.1482	H	7.3259	7.2530	-5.3025
C	3.5078	0.3713	1.2125	H	4.0773	7.0572	-4.5940
H	4.8423	-1.7501	-2.0778	H	1.2947	8.6528	-2.3434
H	5.8385	-3.0922	0.8459	H	3.1989	11.2954	-1.4604
C	4.2920	3.0761	0.5300	H	0.8093	10.1663	0.6172
N	3.5267	0.0645	3.9543	H	6.4982	8.0216	4.0543
H	1.5864	-0.0606	0.5135	H	3.4973	9.5154	4.2671
C	6.7804	3.0205	-0.9490	H	5.9751	10.9976	2.5417
C	9.9733	-0.6432	-2.0012	H	10.7253	-2.4341	-1.2329
H	8.6516	0.5350	1.6421	H	9.2889	-1.0349	-3.9349
C	6.2257	3.5440	-3.7909	H	11.5734	0.6927	-2.1469
C	8.3351	5.3646	-0.2369	H	4.4475	1.3835	5.0152
C	3.0799	5.2218	1.1775	C	2.5457	-1.9172	5.2646
C	6.5884	7.4706	-1.2416	C	1.3483	-3.9741	4.0117
H	10.1657	5.3889	-1.2472	C	0.3801	-6.0153	5.3443
H	8.7317	5.4756	1.8051	C	0.5608	-6.1359	7.9882
C	0.6016	5.2059	2.6020	C	1.6842	-4.1275	9.2528
C	4.2431	7.8154	0.5257	C	2.6427	-1.9886	7.9838
C	5.1064	9.1502	2.9912	H	1.2135	-3.9643	1.9548
C	2.2713	9.5651	-0.7433	H	-0.5122	-7.5639	4.3001
H	7.5597	9.3136	-1.3512	H	-0.1472	-7.7727	9.0309
C	5.9590	6.4695	-3.9353	H	1.8725	-4.2674	11.3114
H	-0.0635	3.2772	3.0000	C	3.7526	0.1634	9.4128
H	-0.8943	6.1886	1.5229	O	5.0104	1.8406	8.4750
H	0.7599	6.1924	4.4382	O	3.3079	0.2603	11.9338
H	4.5203	2.5537	-4.4660	H	2.0533	-0.9857	12.4299
